# Successful recruitment, survival and long-term persistence of eastern oyster and hooked mussel on a subtidal, artificial restoration reef system in Chesapeake Bay

**DOI:** 10.1371/journal.pone.0204329

**Published:** 2018-10-15

**Authors:** Romuald N. Lipcius, Russell P. Burke

**Affiliations:** Virginia Institute of Marine Science, College of William & Mary, Gloucester Point, Virginia, United States of America; Bigelow Laboratory for Ocean Sciences, UNITED STATES

## Abstract

Restoration efforts with native eastern oyster, *Crassostrea virginica*, in Chesapeake Bay and elsewhere have been limited by shell availability, necessitating the use of alternative structures as subtidal reefs, yet these have rarely been evaluated quantitatively. We quantified population structure, density, abundance and biomass of eastern oyster and hooked mussel, *Ischadium recurvum*, on a concrete modular reef (75 m^2^ surface area over 5 m^2^ of river bottom) deployed subtidally at 7 m depth in the Rappahannock River, Virginia during October, 2000. After nearly 5 y (May 2005), we took 120 stratified random samples over the reef. The reef was heavily colonized by 28-168 oysters and 14-2177 mussels m^-2^ surface area. These densities translate to 1085 oysters and 8617 mussels m^-2^ river bottom, which are the highest recorded for artificial oyster reefs. Size structure of oysters reflected four year classes, with over half of oysters more than 1 y old and of reproductive age. Oyster biomass (1663 g dry mass m^-2^ river bottom) and condition index were equally high, whereas parasite prevalence and intensity were low. Oyster density correlated positively in a sigmoid fashion with mussel density up to high densities, then declined. This modular reef is one of the most successful artificial reefs for eastern oyster and hooked mussel restoration, and details features that are conducive for successful settlement, growth and survival in subtidal habitats.

## Introduction

Native oyster reefs have been functionally extirpated worldwide [1, 2]. For instance, in North America the native eastern oyster (*Crassostrea virginica*) along the Atlantic and Gulf of Mexico coasts and Olympia oyster (*Ostrea lurida*) along the Pacific coast have declined by 88% in biomass and by 64% in reef area over the past 200 y [2]. In Chesapeake Bay, the eastern oyster has been reduced to approximately 1% of its previous abundance due to overfishing and oyster reef degradation [3, 4]. These population declines have stimulated considerable restoration efforts, particularly in the Gulf of Mexico and estuaries along the Atlantic coast.

Unfortunately, the preferred substrate for oyster restoration, natural oyster shell, has become a limiting resource, which has spurred the use of alternative reef substrates such as concrete structures. European countries have been experimenting with various types of subtidal artificial reefs over the past few decades [5–9]. Often such reefs serve a dual purpose, such as combined fish and bivalve habitat, and many of these reefs have enhanced commercial harvests, especially of bivalves. Other benefits of these reefs are protection from illegal trawling and of biodiversity. These reefs have demonstrated that alternative reef structures providing the stability and complexity of natural reefs can lead to higher abundance, biomass and diversity of species under restoration.

To ensure that effort and funds are expended judiciously for restoration, credible evaluation of alternative reef structures is critical. For subtidal shell and intertidal restoration reefs, there is ample evidence of their productive performance in restoring the eastern oyster [10–14]. In contrast, subtidal artificial oyster reefs have rarely been evaluated comprehensively [15–17]. Consequently, we provide one of the few exhaustive evaluations of experimental, subtidal artificial reef modules for the eastern oyster. In this study, we document density, abundance, biomass and size structure of the eastern oyster and hooked mussel *Ischadium recurvum* as a function of various features of the artificial reef, which can serve as a model for the use, performance and monitoring of artificial reefs in restoration efforts.

## Materials and methods

### Modular reef design, location and placement

**Fig 1:**
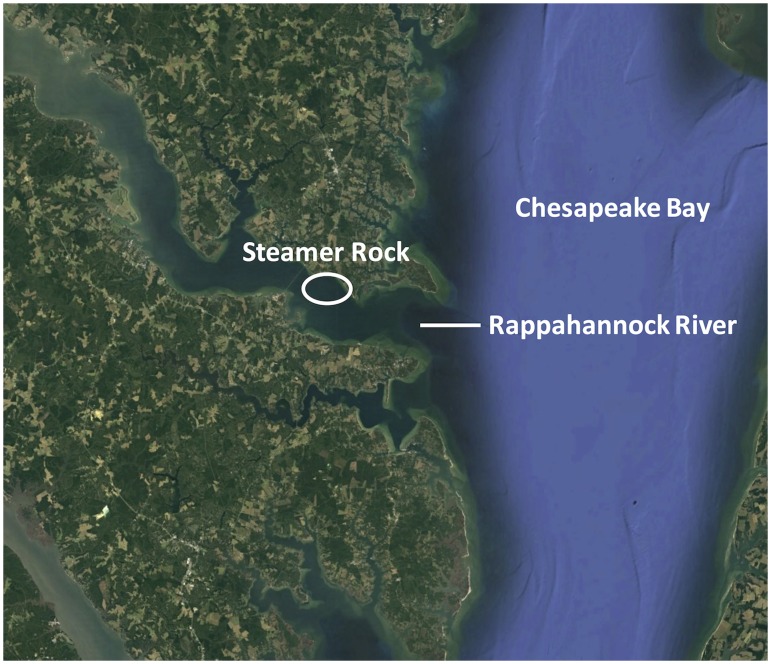
Location of the modular reef. Modular reef was deployed subtidally in 7 m depth at Steamer Rock in the Rappahannock River, Virginia in October 2000 and sampled in late May 2005.

**Fig 2:**
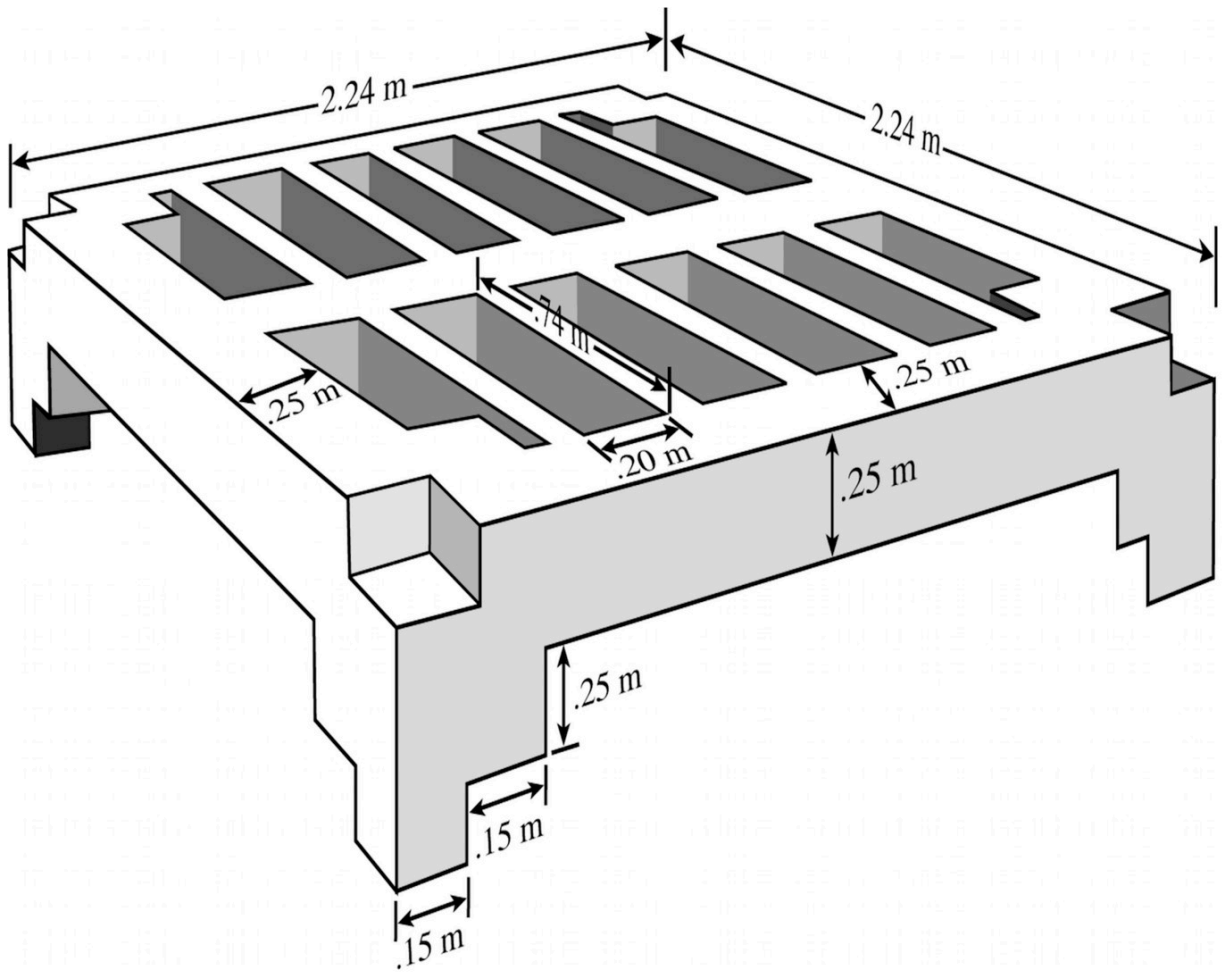
Schematic design of a single module. Total surface area of each module was 74.17 m^2^, with 3.11 m^2^ each on the Top and Bottom faces, 2.89 m^2^ on the Side faces, and 5.73 m^2^ on the Hole faces. Five modules were stacked to comprise one modular reef. Schematic was based on dimensions provided by Retired Naval Captain Robert Jensen (deceased) and McLean Construction Company.

In October 2000, a substantial rebar-reinforced concrete modular reef system was deployed subtidally at 7 m depth near the mouth of the Rappahannock River, a western-shore tributary of Chesapeake Bay (Fig 1). The designer, a retired engineer for the United States Navy (Captain Robert Jensen), intended to provide suitable substrate for eastern oyster in a high-flow, low-siltation habitat. The modular reef was located at Steamer Rock in the Rappahannock River (Fig 1), and consisted of five modules stacked on each other, with four faces (top, side, hole, bottom) per module (Fig 2).

### Sampling procedure and design

**Fig 3:**
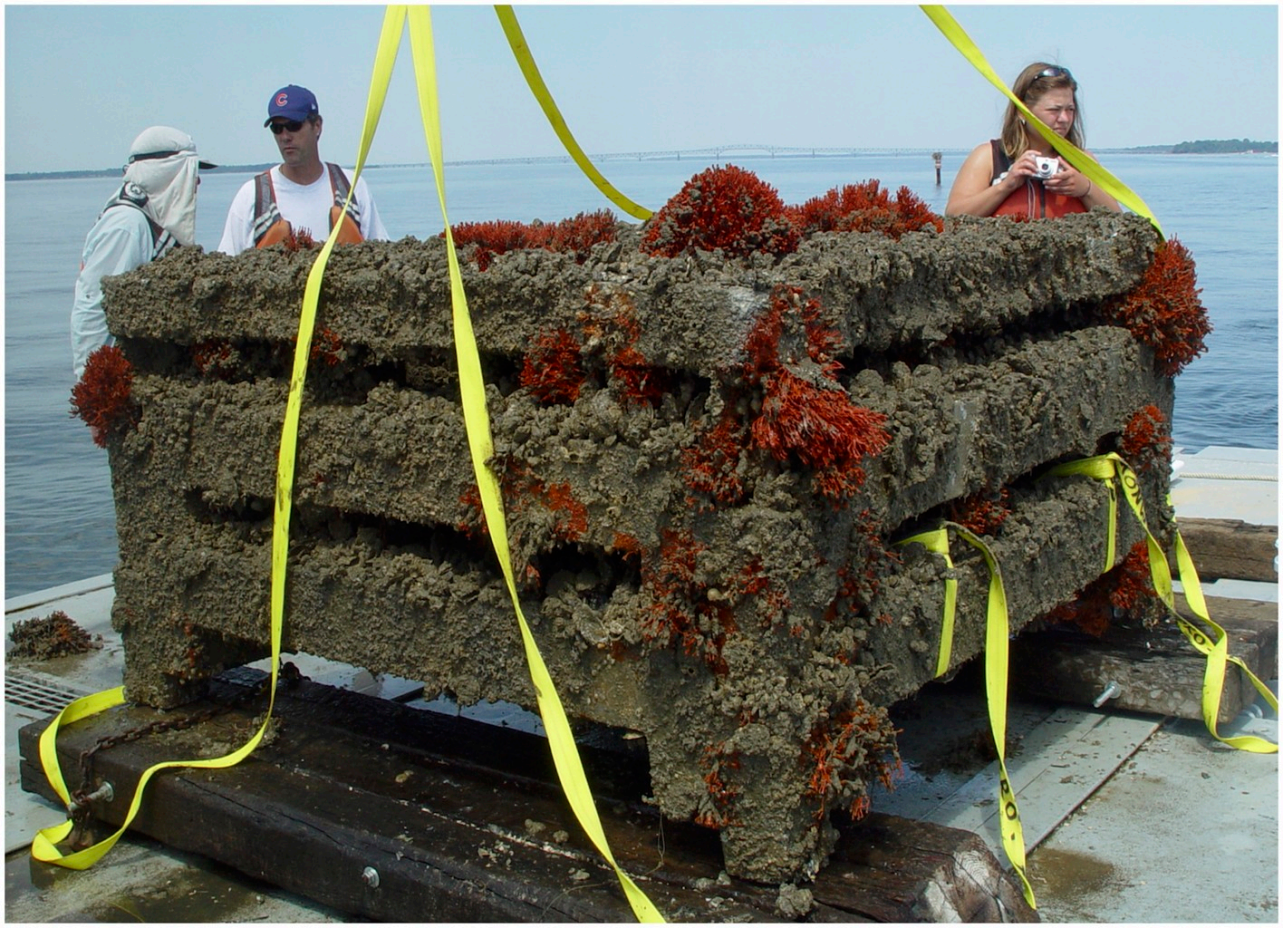
Top three modules of the modular reef that were sampled and immediately returned to the same location. Individuals in the photo are, from left to right, Captain R. Jensen, R. Lipcius, and K. Knick. Photo by L. Latane.

Due to logistical constraints, we were only able to sample the top three modules of the modular reef. However, a commercial diver indicated that the lowest two modules appeared equivalent in oyster and mussel abundance to the upper three. The three modules were secured simultaneously with straps by a commercial diver and brought to the surface by a crane aboard a commercial barge for sampling (Fig 3). To access all faces on each module, the crane on the commercial barge lifted one module off the lower module until all samples were collected. Upon completion, the layers were stacked in the same order on board the barge and returned to the same location at Steamer Rock. Documentation of the reef recovery through photography and videography, and sampling were completed in one day (27 May 2005).

Permission to sample the reef was given by United States Navy Captain (Retired) Robert W. Jensen (deceased). The field sampling did not involve endangered or protected species. The two living individuals (R. N. Lipcius, K. E. Knick) in one of the manuscript photos have given written informed consent (as outlined in PLOS consent form) to publish the photo. The third individual (R. W. Jensen) is deceased but gave verbal permission.

The modular reef was sampled using a stratified random sampling design [18]. Two stratum types were defined, Module and Face. All potential sample plots for each module-face combination were calculated and actual sample plots selected using a random number generator in Microsoft Excel. Each sample plot was defined using a 0.254 m x 0.254 m quadrat with surface area of 0.065 m^2^. When the quadrat did not fit on a particular sample plot, as for plots in the holes of the module, we measured the actual area of the sample. A total of 120 samples was collected; 10 samples were taken from each of the 12 module-face combinations.

Upon removal of the three modules, it became apparent that the lifting straps had removed epifauna at each strap-reef interface. Sample plots that were impacted by the straps were discarded and the next random plot selected. Epifauna were removed from each plot with hand scrapers, placed in large trays, stored in large freezer bags on ice, and transported to the laboratory for processing.

### Laboratory processing: Density, biomass and condition index

Samples were processed in increments of 24 samples (3 modules x 4 faces x 2 replicates). Each sample was thawed and rinsed over a 1-mm mesh sieve. Bivalve (oyster and mussel) and sponge volume were measured using volumetric displacement. Shell height (SH), width, and depth were measured for all bivalves, living and dead. For oysters, SH was considered as the distance from the umbo to the farthest end of the shell.

All internal tissues were collected for each oyster in pre-weighed aluminum weigh boats for dry mass (DM) and ash-free dry mass (AFDM) measurements. All 108 oysters and 138 of the 924 mussels from the first 24 samples were processed for DM and AFDM. The oysters and selected mussels represented the full range of SH values. The DM data for oysters and mussels were used in a length-weight regression to estimate biomass over the entire five-module reef, assuming the size structure produced from all 120 samples was consistent with the size structure produced from the first 24 samples.

Condition Indices [19] were calculated for most of the 108 oysters. All oysters were cleaned of fouling organisms and washed with tap water. After cleaning, oysters were blotted dry before being measured. Measurements made on each oyster included total mass and wet shell mass to the nearest 0.001 g, and SH to the nearest 0.1 mm. After shucking, shells and tissue were dried at 60°C for at least 48 h and weighed. Condition Index (CI) was calculated using three equations:

$$\begin{array}{c} CI_{1}=\frac{Dry\;Tissue\;Weight}{Shell\;Cavity\;Volume}\times 100 \end{array}$$ (1)

$$\begin{array}{c} CI_{2}=\frac{Dry\;Tissue\;Weight}{Dry\;Shell\;Cavity\;Volume}\times 100 \end{array}$$ (2)

$$\begin{array}{c} CI_{3}=\frac{Dry\;Tissue\;Weight}{Dry\;Shell\;Weight}\times 100 \end{array}$$ (3)

with *CI*_1_ [20], *CI*_2_ [21], and *CI*_3_ [22].

For *CI*_1_ and *CI*_2_, shell cavity volume is equal to the difference between the mass of the whole oyster (g) and the mass of the empty valves (g) [20, 21]. *CI*_1_ considered the mass of the empty shells immediately after shucking whereas *CI*_2_ used the mass of the shells after a period of drying [20, 21]. For all analyses, condition indices were used where shell volume was calculated by a gravimetric method. These measures are linearly related to those where CI is calculated by a volumetric method; i.e., by water displacement of the shells [23]. Of the remaining 96 samples, volume was measured as indicated previously.

### Population structure

We analyzed size and age structure for all 120 oyster samples (523 oysters), whereas for mussels we only described size structure, using 24 of the 120 samples (924 mussels). To determine size- and age-class peaks of eastern oyster, we first defined the number of annual cohorts for 2001 through 2004 from spatfall and benthic samples [24–27], and growth rates for oysters in the Rappahannock River and two tributaries immediately north (Great Wicomico River) and south (Piankatank River) of the Rappahannock River [28–30]. The spatfall data indicated that there could be one or two annual peaks (cohorts) y^-1^ in spatfall. Based on the initial estimates of spatfall, oyster size and growth, we developed five potential cases for the number of cohorts for the 2001 through 2004 age classes (Table 1): (i) one cohort y^-1^ in all years; (ii) one cohort y^-1^ for 2001 through 2003, and two cohorts in 2004; (iii) one cohort y^-1^ in 2001 and 2003, and two cohorts y^-1^ in 2002 and 2004; (iv) one cohort in 2003, and two cohorts y^-1^ in 2001, 2002, and 2004; and, (v) one cohort each in 2001, 2002 and 2004, and none in 2003. We eliminated the 2003 cohort in case (v) because spatfall and oyster abundance were extremely low in 2003 [26]. We did not analyze mussel size- or age-structure because basic information on age, size and growth of hooked mussel were unavailable in the published literature.

**Table 1:** Characteristics of the five potential cohort structures for eastern oyster examined with the R package mixtools. There were four year classes with either one or two cohorts, except in 2003 when spatfall and oyster abundance were too low to distinguish more than one cohort. Nominal = starting values entered into mixtools for mean size of each cohort; *μ*, *σ* and proportion = estimates of cohort mean size, standard deviation, and proportion of the population in each cohort generated by mixtools.

Number of cohorts	Estimates	Annual cohorts						
		2004		2003	2002		2001	
		Jul	Aug	Jul	Jul	Aug	Jul	Aug
7	nominal	20	45	55	80	90	100	110
	*μ*	21.9	43.6	61.7	78.3	89.3	91.1	100.5
	*σ*	7.2	3.5	0.8	7.8	1.5	17.8	3.7
	proportion	0.36	0.05	0.02	0.14	0.04	0.34	0.06
6	nominal	20	40	70	90	100	115	
	*μ*	21.8	43.6	61.6	85.2	102.3	118.2	
	*σ*	7.2	3.7	0.7	13.0	3.0	8.5	
	proportion	0.36	0.05	0.01	0.49	0.04	0.04	
5	nominal	25		55	85	110	130	
	*μ*	21.9		43.6	85.1	102.3	119.4	
	*σ*	7.2		3.3	14.1	2.9	8.2	
	proportion	0.36		0.05	0.52	0.04	0.03	
4	nominal	15		45	90		110	
	*μ*	21.7		67.3	89.9		121.8	
	*σ*	7.1		25.5	12.8		4.7	
	proportion	0.34		0.23	0.42		0.02	
3	nominal	25			70		100	
	*μ*	21.9			43.5		88.0	
	*σ*	7.2			3.2		16.3	
	proportion	0.36			0.04		0.59	

The five cases were used along with the respective nominal mean size of each cohort (Table 1) in the R package “mixtools” [31, 32], assuming a mixture of Gaussian distributions, to generate estimates of mean size and variance for each cohort. The cases were also evaluated in mixtools assuming a mixture of gamma distributions, but these either did not converge or they generated unrealistic estimates. When the models converged and generated realistic estimates, the log-likelihood estimates for each case were used to calculate corrected AIC (Akaike Information Criterion) values and weighted probabilities [33] to select the best-fitting model.

### Parasite prevalence and intensity

Thirty large oysters (75.6-125.2 mm SH) were haphazardly sampled from the different module faces for pathology tests performed within 2 weeks of sampling. Samples were brought back live on ice to the Pathology Laboratory at VIMS. Presence and concentration of Dermo *Perkinsus marinus* and MSX *Haplosporidium nelsoni* were determined. Some other parasites and pathogens commonly found in oyster tissue, but not generally associated with serious disease and mortality, were noted. These included *Nematopsis*, *Rickettsia*-like organisms, *Sphenophyra*-like ciliates, *Stegotricha* ciliates, and viral gametocytic hypertrophy.

## Results

### Density

**Fig 4:**
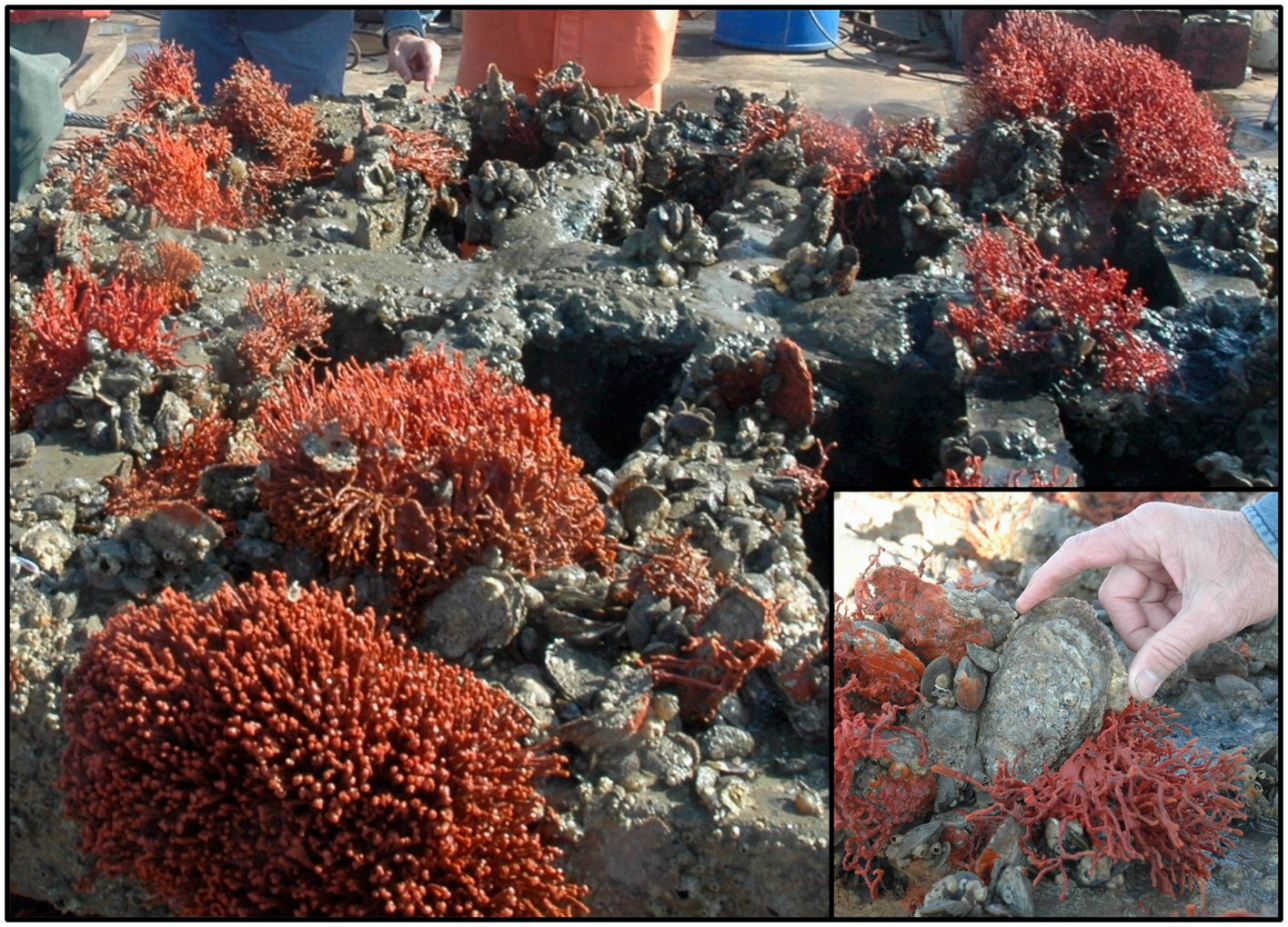
Top layer of one module. Visible are oyster clusters, hooked mussels and redbeard sponges *Clathria prolifera*, which are characteristic of a healthy subtidal oyster reef. Inset: closeup of a large, live eastern oyster of approximately 130 mm shell height.

The modular reef was heavily colonized by eastern oysters of multiple year classes, hooked mussels, and redbeard sponges (Fig 4). Oysters and mussels recruited and survived at densities m^-2^ reef surface area ranging from 28-168 and from 14-2177, respectively.

**Fig 5:**
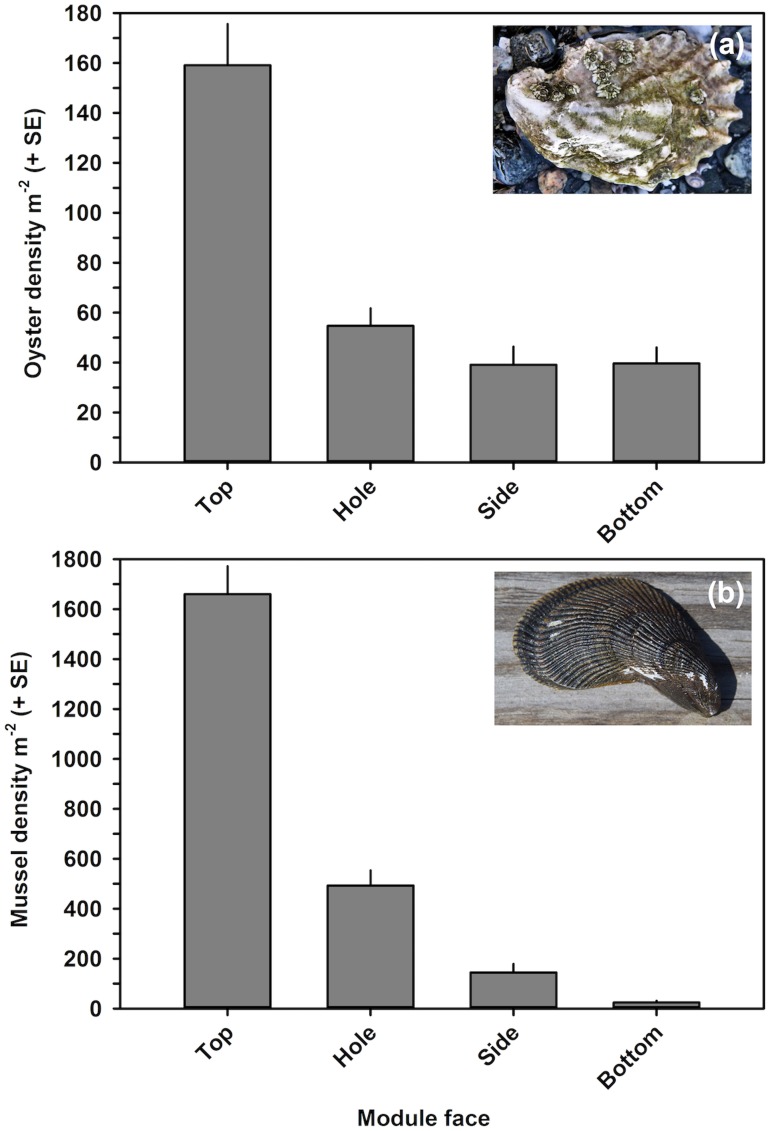
Live oyster density and mussel density. Density m^-2^ by face of the modular reef.

Oyster and mussel density were analyzed using four linear models with Module and Face as fixed factors (Table 2). For oyster density, the interaction and Module-only models were rejected due to low AIC weighted probabilities (Table 2). The Face-only model was significantly better than the additive model (Log-likelihood *X*^2^ test, *p* < 0.0001), the factor Face was significant (ANOVA, *p* < 0.0001), and it was selected as the best-fitting model. Oyster density was significantly higher on the top Face, at 159.1 individuals m^-2^ surface area, than on all other faces (Fig 5; Tukey HSD test, *p* < 0.0001), which did not differ significantly from each other (Tukey HSD test, *p* > 0.70) and whose average densities were below 60 individuals m^-2^ surface area (Fig 5). The mean fraction of dead oysters (Dead oyster density/Total oyster density) was 0.31, and did not differ significantly by either Module or Face (ANOVA, *p* > 0.156).

**Table 2:** Results of the AIC analysis for live oyster density, mussel density, and oyster biomass m^-2^. In all three cases, the best-fitting model provided a significantly better fit than all other models (Log-likelihood *X*^2^ test, *p* < 0.0001).

Model	k	AIC_*c*_	Δ_*i*_	*w*_*i*_
*Oysterdensity*				
*Module* x *Face*	13	1327.3	14.6	<0.01
*Module* + *Face*	7	1317.1	4.3	0.10
*Module*	4	1382.9	70.1	<0.01
*Face*	5	1312.7	0	0.90
*Musseldensity*				
*Module* x *Face*	13	1698.8	0	1.00
*Module* + *Face*	7	1746.0	47.2	<<0.001
*Module*	4	1929.6	230.8	<<0.001
*Face*	5	1760.3	61.5	<<0.001
*Oysterbiomass*				
*Module* x *Face*	13	1289.6	13.2	0.001
*Module* + *Face*	7	1278.8	2.4	0.22
*Module*	4	1283.3	6.9	0.02
*Face*	5	1276.4	0	0.75

For mussel density, the only model fitting the data was the interaction model; all others had weighted probabilities << 0.001 (Table 2). The interaction model was significantly better than all other models (Log-likelihood *X*^2^ test, *p* < 0.0001), the interaction effect Module x Face was significant (ANOVA, *p* < 0.0001), and it was selected as the best-fitting model. Irrespective of module, mussel density was significantly higher on the Top face, at 1660.1 individuals m^-2^ surface area, than on all other faces (Fig 5; Tukey HSD test, *p* < 0.001). Across all modules, mussel density was higher on Hole faces (492.8 individuals m^-2^) than on Side and Bottom faces (144.6 and 24.3 individuals m^-2^, respectively), and significantly so in three of six cases (Tukey HSD test, *p* < 0.05). Mussel density on Side and Bottom faces did not differ significantly for any module (Tukey HSD test, *p* > 0.9). The mean fraction of dead mussels (Dead mussel density/Total mussel density) was low with a mean of 0.06, and was usually significantly higher on the Top face than on the other three faces (Tukey HSD test, *p* < 0.05).

To compare oyster and mussel densities with those of other alternative and natural oyster reefs, we calculated densities per m^2^ of river bottom for both a single module and for the modular reef composed of five stacked modules (Table 3). For a single module, oyster and mussel densities averaged 208.2 and 1690.1 individuals m^-2^ river bottom, respectively (Table 3). When calculated over the five-module modular reef, these translated to 1041.1 oysters and 8450.4 mussels m^-2^ river bottom (Table 3). To allow fishery managers to assess oyster densities as a function of oyster fishery categories, we also calculated oyster density by spat/recruit, seed/sublegal, and market/legal categories (Table 4).

**Table 3:** Live oyster and mussel density standardized to (i) surface area of each face (density m^-2^ surface area, (ii) density m^-2^ river bottom on each module, which is a product of the density m^-2^ surface area and total surface area per face on a module, and (iii) density m^-2^ river bottom on the five-module modular reef. Total surface area of each module was 14.84 m^2^, with 3.11 m^2^ each on the Top and Bottom faces, 2.89 m^2^ on the Side faces, and 5.73 m^2^ on the Hole faces.

Face	Density					
	Module surface (m^-2^ surface area)		Module (m^-2^ river bottom)		Modular reef (m^-2^ river bottom)	
	Oyster	Mussel	Oyster	Mussel	Oyster	Mussel
*Top*	159.1	1660.1	98.6	1029.0	493.1	5144.8
*Hole*	54.7	492.8	62.5	562.8	312.5	2813.8
*Side*	39.1	144.6	22.5	83.3	112.6	416.3
*Bottom*	39.7	24.3	24.6	15.1	122.9	75.4
*Total*			208.2	1690.1	1041.1	8450.4

**Table 4:** Live oyster density, standardized to density m^-2^ river bottom on the five-module modular reef, for the typical categories in oyster fisheries.

Category	Size class (shell height)	Percentage	Density (m^-2^ river bottom)
*Spat*/*Recruit*	<25 mm	24.6%	256 m^-2^
*Seed*/*Sublegal*	25-75 mm	28.5%	296 m^-2^
*Market*/*Legal*	>75 mm	46.9%	489 m^-2^

In terms of the distribution of oysters by face over 1 m^2^ of river bottom (Table 3), Top faces only composed 21.0% of each module’s surface area, yet they harbored 47.3% of all oysters. Hole faces held 30.0% of oysters, which was somewhat lower than their surface area of 38.6%. Side and bottom faces composed 19.5 and 21.0% of area, but had only 10.8 and 11.8% of oysters, respectively. For mussels, Top faces also harbored the highest fraction at 60.9% of all mussels despite only having 21.0% of each module’s surface area (Table 3). Hole faces held 33.3% of mussels over 38.6% surface area. Side and Bottom faces were impoverished in mussel density, holding only 4.9 and 0.9% of all mussels, respectively.

**Fig 6:**
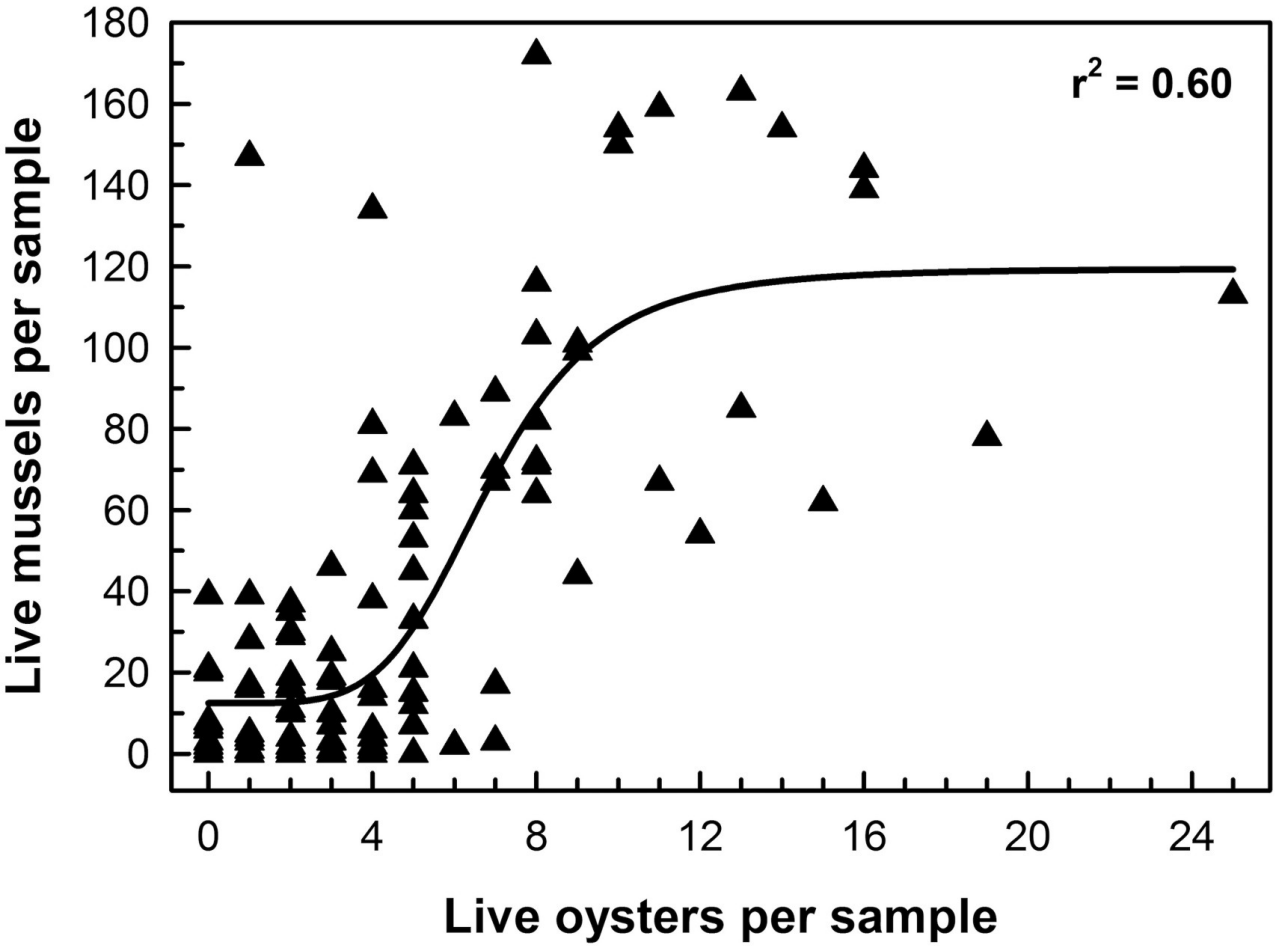
Relationship between live mussel density and oyster density. The curve is sigmoid and statistically significant (Nonlinear least squares regression, SigmaPlot 13, *p* < 0.0001).

Oyster density and mussel density were significantly and positively correlated with a sigmoid relationship such that mussel density increased exponentially with oyster density and then reached an asymptote at high oyster density (Fig 6).

### Population structure

**Fig 7:**
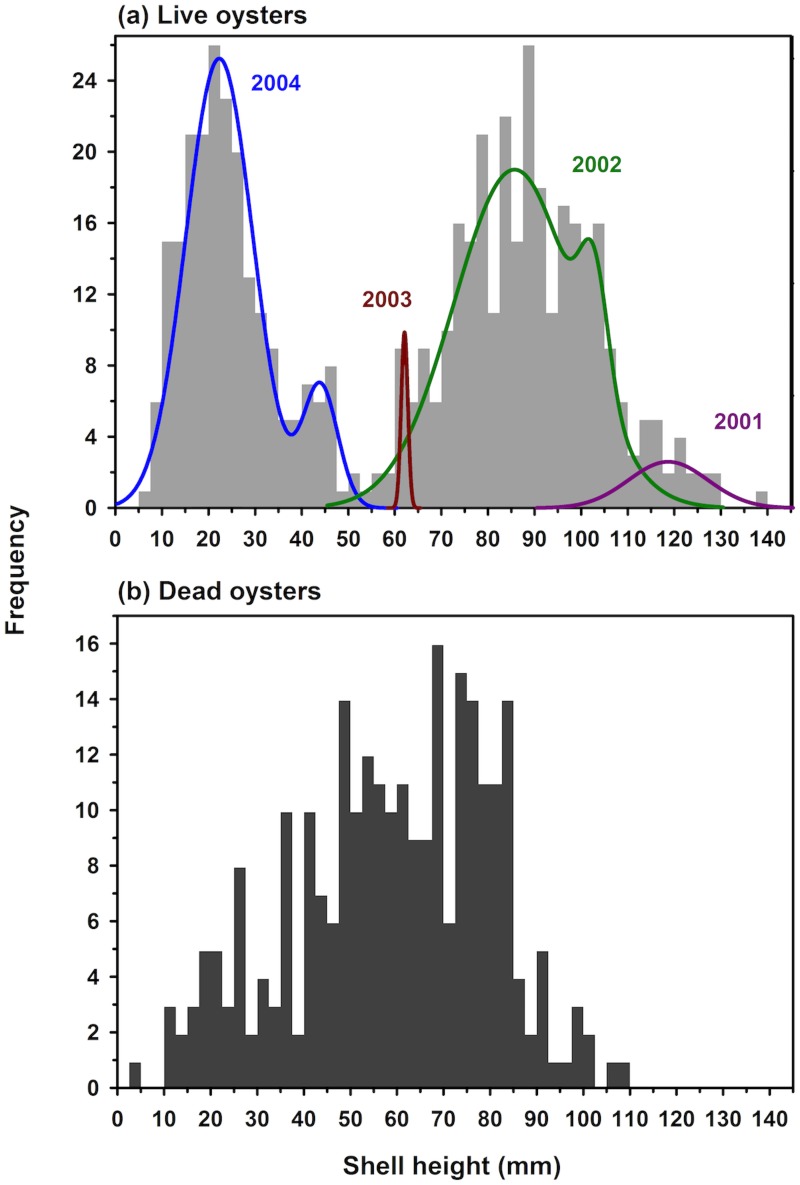
Size frequency of live (a) and dead (b) oysters on the modular reef. The cohorts were distinguished using the R package mixtools. Cohort parameters are in Table 1.

**Fig 8:**
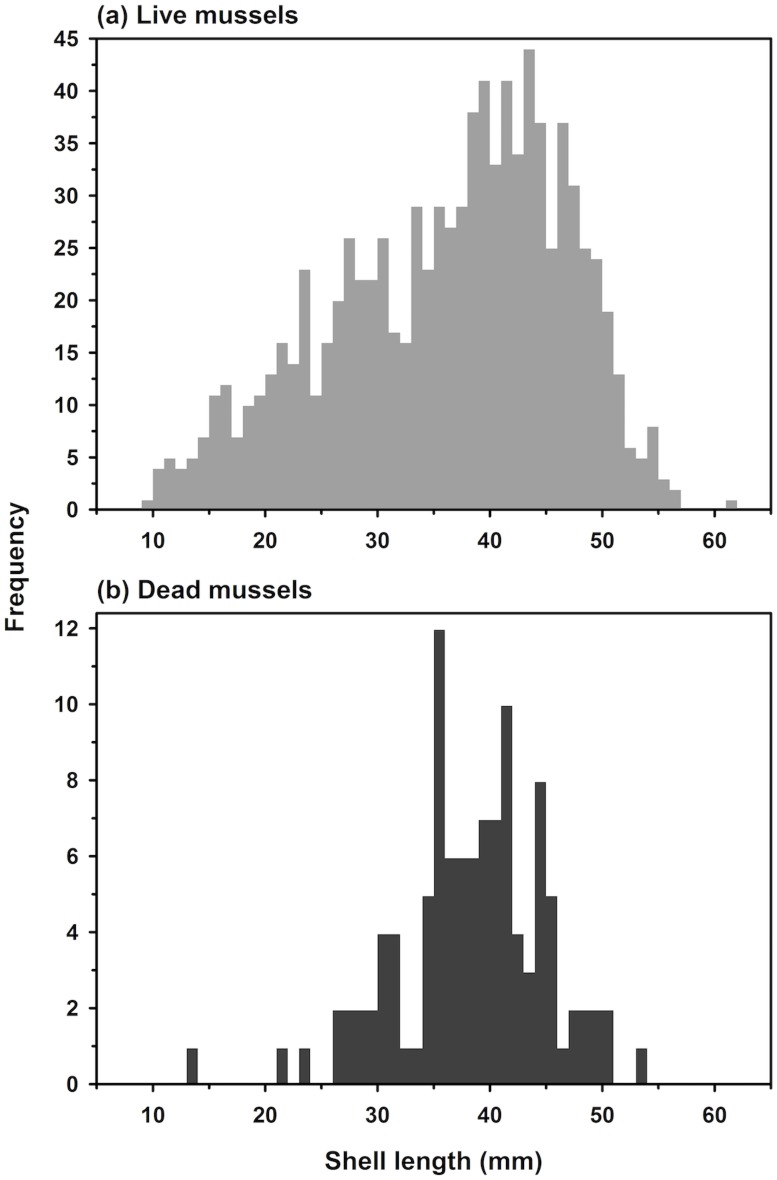
Size frequency of live (a) and dead (b) mussels on the modular reef.

Of the possible 523 oysters, 520 were used in the size- and age-structure analysis (Table 1, Fig 7a). Oyster SH ranged from 7.1 to 139.0 mm, with a maximum of four year classes (2001-2004) since the reef was deployed in 2000 at the end of the settlement season and sampled in May 2005 before the 2005 settlement season. Of the five possible cases representing four year classes (Table 1), only the cases with five or six cohorts across four year classes were realistic and converged (Table 5). Of those, the case with six cohorts fit the data significantly better than the case with five cohorts (Log-likelihood *X*^2^ test, *p* < 0.0001). Approximately 58% of all oysters were 1+ y of age (53% in 2001, 4% in 2002, and 1% in 2003 year classes) and reproductive (Table 1, Fig 7a). The 2003 year class was largely missing (1%), while the age 0 year class of 2004 represented 41% of all oysters. The size structure of hooked mussel was a conglomeration of several intermixed year classes spanning the range of mussel SHs from 9.2 to 61.0 mm SL (Fig 8a).

**Table 5:** Results of the AIC analysis for size structure of live oysters using the only two realistic models, both with four year classes, and one with five and the other with six cohorts. The best-fitting model with six cohorts provided a significantly better fit (Log-likelihood *X*^2^ test, *p* < 0.0001).

Modeled cohorts	k	Log likelihood	AIC_*c*_	Δ_*i*_	*w*_*i*_
5	10	-2401.64	4823.7	5.14	0.07
6	12	-2396.97	4818.6	0	0.93

The numbers of dead oysters and mussels were low and their size structures (Figs 7b and 8b) did not mirror those of live oysters and mussels (Figs 7a and 8a). In analyzing size structure of dead bivalves, we assume that there may be a bias against the preservation of small individuals, so we limit our assessment to dead oysters > 20 mm SH and mussels > 20 mm SL. In addition, we assume that much of the mortality of oysters and mussels occurred in summer and fall such that the dead oysters we sampled in May were smaller in size than their living age-class counterparts, which grew during the preceding fall and spring before May sampling.

For oysters, comparison of live and dead oyster size structures indicated that (i) many older (ages 2 and 3), larger oysters survived and likely produced some oysters living to 5 or 6 y, and (ii) a high proportion of mortality occurred for age 2 and 3 oysters, but not as much for age 0 oysters once they had reached > 20 mm SH (Fig 7b). Similarly, for mussels the size structure comparison demonstrated that mussels also would have survived to be 5 or 6 y old, and that most mortality occurred when mussels were 2 and 3 y of age (Fig 8b).

### Biomass

**Fig 9:**
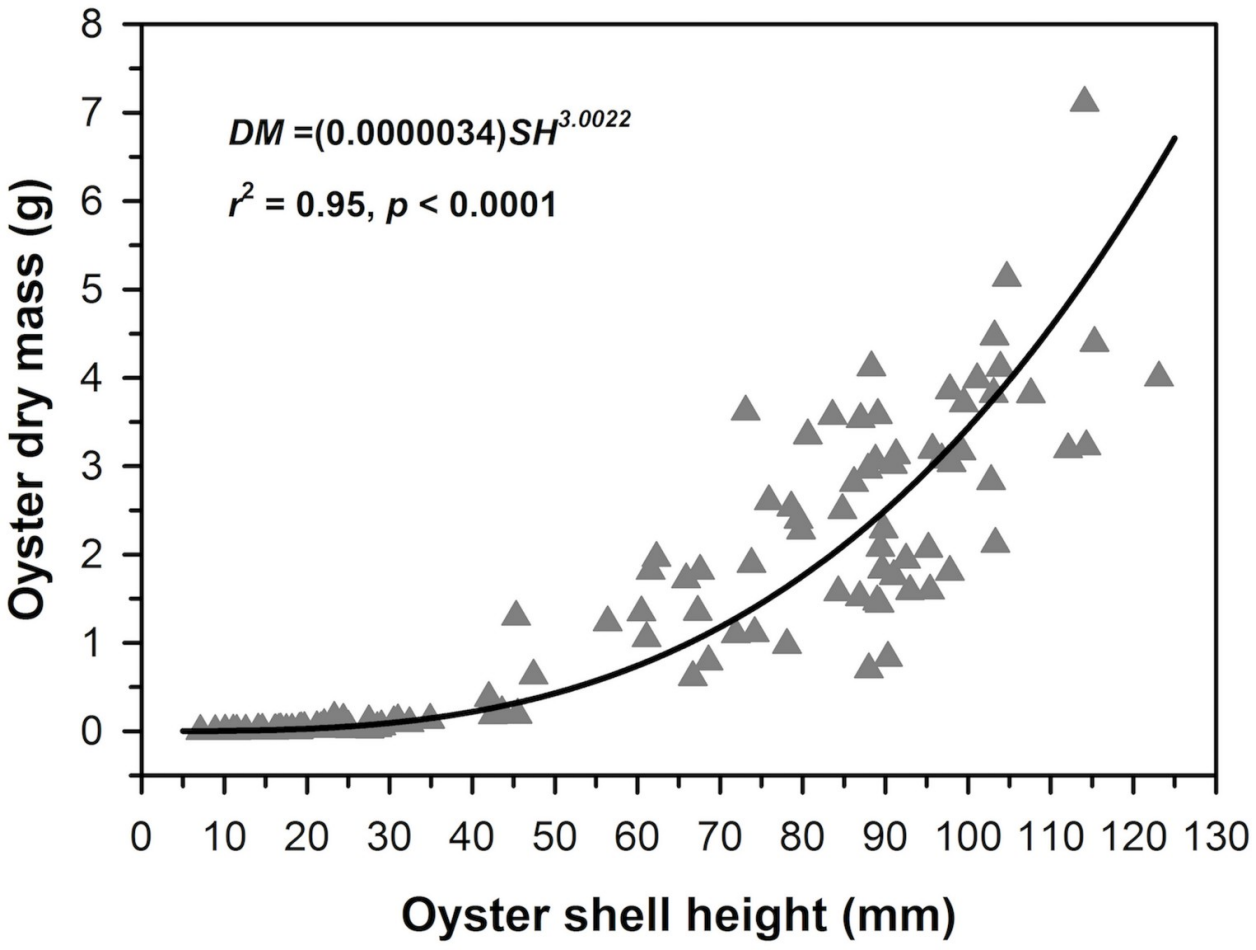
Live oyster DM as a function of SH.

Biomass was estimated for oysters using linear regression of log_10_transformed dry mass (DM) as a function of log_10_transformed shell height of oysters. The regression was highly significant (Fig 9); the equation was used to calculate DM of oysters for the analysis of biomass.

**Fig 10:**
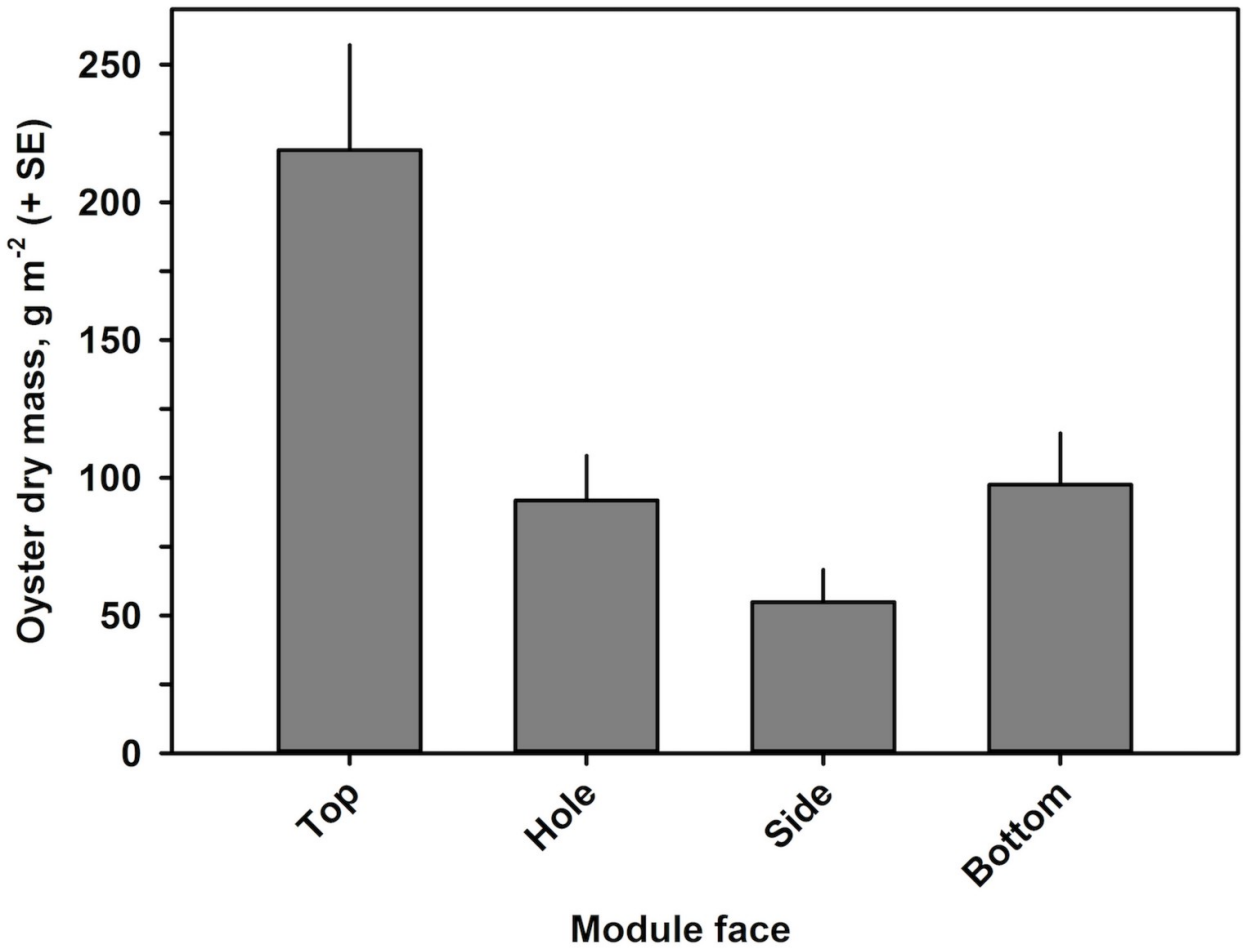
Live oyster biomass, as dry mass (DM), by face of the modular reef.

Oyster DM was also analyzed using four linear models with Module and Face as fixed factors (Table 2). Similar to oyster density, the interaction and Module-only models of oyster DM were rejected due to low AIC weighted probabilities (Table 2). The Face-only model was significantly better than the additive model (Log-likelihood *X*^2^ test, *p* < 0.0001), the factor Face was significant (Gamma generalized linear model, *p* < 0.005), and it was selected as the best-fitting model. Similar to the results for oyster density, DM was significantly higher on the Top face, with 219.0 g DM m^-2^ surface area, than on all other faces (Fig 10; Gamma generalized linear model, *p* < 0.005). In contrast to oyster density, the Bottom and Hole faces had similarly high DM values of 97.5 and 91.8 g DM m^-2^ surface area, respectively, and which did not differ significantly (Gamma generalized linear model, *p* > 0.80). DM on the Side face was 54.9 g DM m^-2^ surface area and significantly lower than that on all other faces (Gamma generalized linear model, *p* < 0.05).

As with oyster and mussel density, we calculated oyster biomass in g per m^2^ of river bottom for both a single module and for the modular reef (Table 6). Per module, oyster biomass averaged 332.6 g DM m^-2^ river bottom (Table 6). When calculated over the five-module modular reef, this translated to 1663.1 g DM m^-2^ river bottom (Table 6).

**Table 6:** Oyster biomass, as dry mass (DM), by module-face stratum combination.

Face	Module surface (g DM m^-2^ surface area)	Module (g DM m^-2^ river bottom)	Modular reef (g DM m^-2^ river bottom)
*Top*	219.0	135.7	678.7
*Hole*	91.8	104.8	524.2
*Side*	54.9	31.6	158.1
*Bottom*	97.5	60.4	302.2
*Total*		332.6	1663.1

The distribution of oyster biomass by face over 1 m^2^ of river bottom differed somewhat from that for density (Table 3). Hole faces held 31.5%, Side faces 9.5%, Top faces 40.8%, and Bottom faces 18.2% (Table 6).

### Condition index

Sixty-two oysters throughout the full size range were processed to produce the three CIs (Eqs 1 to 3). There was no significant difference between any of the CIs as a function of face or module (ANOVA, *p* > 0.1). The mean CI values for CI1 to CI3 were 12.2, 8.8 and 6.0, respectively, which are all near the upper end of reported CI values [22].

### Parasite prevalence and intensity

Of the 30 large oysters processed for parasite assessment, none was infected with MSX and 30% were infected with Dermo. Of the nine oysters infected with Dermo, none had a serious infection (four infections were light and five were rare). The following pathogens were found in one or more oysters: *Nematopsis* (1), *Rickettsia*-like organisms (1), *Sphenophyra*-like ciliates (11), *Stegotricha* spp. ciliates (1), and viral gametocytic hypertrophy (1).

## Discussion

The key findings of our study, which we believe is one of the most thorough evaluations of subtidal artificial oyster reefs [16, 17], relate to reef features that are most conducive for successful settlement, growth and survival of the eastern oyster and hooked mussel in subtidal habitats.

After nearly 5 y of deployment at 7 m in the Rappahannock River, the reef was heavily colonized by an average of 1041 oysters and 8450 mussels m^-2^ river bottom, which are the highest recorded for subtidal, artificial oyster reefs [16]. Of the oysters, about 41% were age 0 individuals (i.e., spat), while the remaining 59% were reproductive-age 1+ individuals, which translates to averages of 427 age 0 and 614 adult oysters m^-2^ river bottom. These extremely high densities were the product of densities per unit surface area and the total reef surface area per m^2^ of river bottom, which reflected the complex architectural design of the modular reef. By comparison, seven systems of subtidal, artificial oyster reefs constructed of concrete and other similar materials, and deployed throughout the Gulf of Mexico from Alabama through Texas averaged about 15 age 0 and 85 adult oysters m^-2^ [16]. Total average densities on these artificial reefs ranged up to 392 oysters m^-2^ and on nearby shell reefs up to 611 m^-2^ [16]. In contrast, total oyster densities on intertidal artificial reefs in the Gulf of Mexico averaged up to 3200 oysters m^-2^ [34], though these were smaller in size such that biomass may have been comparable across systems.

Oyster densities of spat and adults on the modular reef were comparable to those on the most successful restored shell reefs for the eastern oyster (i.e., high-relief reefs in the Great Wicomico River, Chesapeake Bay, Virginia), which averaged 683 adults and 344 spat m^-2^ [11], and higher than those on other successful restoration shell reefs for eastern oyster [10, 12]. Oyster densities on the modular reef were also comparable to or higher than those on natural, unharvested subtidal reefs in Louisiana and Texas, which averaged as much as 196 age 0 and 147 adult oysters m^-2^ river bottom [35], and on a network of naturally occurring relict oyster reefs in the Lafayette River, a tributary of lower Chesapeake Bay (R. Lipcius, R. Burke, D. Schulte; unpublished data).

In addition, oyster density and mussel density were significantly and positively correlated with a sigmoid relationship such that the modular reef provided suitable habitat for another suspension-feeding bivalve, thereby augmenting ecosystem services of the reef. Hence, this artificial reef is an extremely effective structure for restoring eastern oyster and hooked mussel populations.

Population size structure of eastern oyster on the modular reef also reflected a persisting, successful reef due to its being composed of four year classes, over half of which (59%) were of reproductive age. Biomass of eastern oyster was equally high, and averaged 1663 g dry mass m^-2^ river bottom. Collectively, oyster density, biomass, and age structure far surpassed the targets for successful oyster reef performance developed for Chesapeake Bay restoration reefs, specifically 50 oysters, 50 g dry mass, and multiple year classes m^-2^ river bottom [36].

The fractions of dead oysters and mussels were relatively low at 0.31 and 0.06, respectively. Comparisons of live and dead oyster age structure indicated that many older (1 to 3+ y of age), larger oysters survived, that a high proportion of mortality occurred for age 2 and 3 oysters, but that mortality was low for age 0 oysters once they reached > 20 mm SH. Similarly, many mussels survived to 3+ y of age, such that most mortality occurred when mussels were somewhere between 2 and 3 y of age. The notable survival was reflected in the high condition index and low disease incidence, which when combined with exceptional biomass and abundance, indicate that the modular reef is an excellent model of effective artificial reefs for eastern oyster restoration.

Oyster and mussel densities were significantly higher on the horizontal Top face, at 159 and 1660 individuals m^-2^ surface area, respectively. For oysters, all other faces did not differ significantly from each other, with average densities below 60 individuals m^-2^ surface area. Our results are consistent with those of a study in the Gulf of Mexico, which indicated that vertical surfaces were favored over horizontal surfaces by oyster larvae during periods of higher sedimentation, but that the reverse was true when sedimentation was low [37]. Our modular reef was in an area of strong currents, which we believe were sufficient to both deliver food and larvae to all portions of the reef and to reduce sediment buildup on the horizontal Top faces of each module. Moreover, we posit that the high density and low mortality across the entire reef can be credited to both the reef’s innovative design, which maximizes flow through the reef, and the location, which has strong tidal currents. We also suggest that mussel density was highest on the Top face, not only due to the strong currents, but also due to the protection afforded by crevices within oyster clusters [38].

Sampling the separate reef modules on deck of a barge allowed us to detect a substantial portion of oysters and mussels that would not have been easily detected underwater by SCUBA divers. In particular, oysters and mussels inhabiting the Hole and Bottom faces would be very difficult to see underwater, potentially biasing density estimates low by up to 50%. Hence, effective evaluation of structurally complex artificial reefs requires attention to the architectural features of specific reefs.

The modular reef performed exceptionally well in an unstructured habitat dominated by muddy sand. Thus, the gains in secondary production at that site were extremely high and the potential for the reintroduction of critical three-dimensional habitats to areas of low native oyster and hooked mussel production are promising. This increased capacity for metapopulation expansion and the associated ecosystem services, such as improved water clarity, due to filter feeders (e.g., oysters, mussels, barnacles, sponges, tunicates) aligns directly with the needs of native oyster and ecosystem restoration.
